# Targeted RNA *N* ^6^‐Methyladenosine Demethylation Controls Cell Fate Transition in Human Pluripotent Stem Cells

**DOI:** 10.1002/advs.202003902

**Published:** 2021-03-18

**Authors:** Xuena Chen, Qingquan Zhao, Yu‐Li Zhao, Guo‐Shi Chai, Weisheng Cheng, Zhiju Zhao, Jia Wang, Guan‐Zheng Luo, Nan Cao

**Affiliations:** ^1^ The Seventh Affiliated Hospital Zhongshan School of Medicine Sun Yat‐sen University Guangdong 510080 P.R. China; ^2^ Key Laboratory for Stem Cells and Tissue Engineering (Sun Yat‐sen University) Ministry of Education Guangdong 510080 P.R. China; ^3^ MOE Key Laboratory of Gene Function and Regulation State Key Laboratory of Biocontrol School of Life Sciences Sun Yat‐sen University Guangzhou 510275 P.R. China

**Keywords:** ALKBH5, CRISPR, differentiation, m^6^A RNA modification, pluripotent stem cells

## Abstract

Deficiency of the *N* ^6^‐methyladenosine (m^6^A) methyltransferase complex results in global reduction of m^6^A abundance and defective cell development in embryonic stem cells (ESCs). However, it's unclear whether regional m^6^A methylation affects cell fate decisions due to the inability to modulate individual m^6^A modification in ESCs with precise temporal control. Here, a targeted RNA m^6^A erasure (TRME) system is developed to achieve site‐specific demethylation of RNAs in human ESCs (hESCs). TRME, in which a stably transfected, doxycycline‐inducible dCas13a is fused to the catalytic domain of ALKBH5, can precisely and reversibly demethylate the targeted m^6^A site of mRNA and increase mRNA stability with limited off‐target effects. It is further demonstrated that temporal m^6^A erasure on a single site of *SOX2* is sufficient to control the differentiation of hESCs. This study provides a versatile toolbox to reveal the function of individual m^6^A modification in hESCs, enabling cell fate control studies at the epitranscriptional level.

## Graphical abstract

*N* ^6^‐methyladenosine (m^6^A) plays a crucial role in regulating stem cell fate determination. A targeted RNA m^6^A erasure system is developed to achieve site‐specific demethylation of RNAs in human embryonic stem cells (hESCs) and it is further demonstrated that temporal m^6^A erasure on a single site of *SOX2* is sufficient to control the differentiation of hESCs.

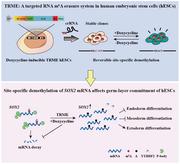

In mammalian cells, regulatory processes at the post‐transcriptional level are often a key determinant of genetic information flow. *N* ^6^‐methyladenosine (m^6^A), as the most abundant modification on messenger RNAs (mRNAs), is involved in nearly all the post‐transcriptional processes, including RNA splicing, processing, transport, as well as RNA stability and translation efficiency.^[^ ^1^ ^]^ The dynamic regulation of m^6^A is reversibly mediated by the methyltransferase complex (“writers”, METTL3/METTL14/WTAP), demethylases (“erasers”, FTO and/or ALKBH5), and many RNA‐binding proteins (“readers”, YTHDF1‐3, YTHDC1/2, and IGF2BP1‐3).^[^ ^1^, ^2^ ^]^ Transcriptome‐wide m^6^A profiling and genetic perturbations of these m^6^A writers, erasers, and readers have linked m^6^A to a wide range of biology and disease processes, like cellular heat shock response,^[^ ^3^ ^]^ spermiogenesis,^[^ ^4^ ^]^ adipogenesis,^[^ ^5^, ^6^ ^]^ and tumorigenesis.^[^ ^7^, ^8^ ^]^

On account of the highly dynamic nature, m^6^A modifications on the pre‐existing RNAs may promote a fast response to external cues during times of cellular transformation or differentiation, thus are particularly important for various development processes and stem cell fate control.^[^ ^9^, ^10^, ^11^, ^12^, ^13^ ^]^ Consistently, genetic ablation of the m^6^A methyltransferase complex induces global reduction of m^6^A abundance in embryonic stem cells (ESCs) and results in blocked differentiation, suggesting a crucial role of m^6^A methylation in regulating early cell fate specification during embryogenesis.^[^ ^14^, ^15^ ^]^ However, such studies are limited by the bulk nature of these experiments in which the methylation levels of thousands of sites are altered, rather than focusing methylation on a single site within a transcript of interest. This raises a pivotal question: are the deficiencies in cell differentiation arising from a single RNA methylation event or an ensemble of m^6^A modifications of multiple sites that function as a synergistic unit? To interrogate the site‐specific effect of m^6^A, introducing point mutation to permanently remove the modification site is a common methodology. However, it is not suitable for cell differentiation studies for the highly dynamic property of m^6^A, and the altered genetic codes may induce unintentional artificial consequences. The absence of a reversible and controllable system that allows for regional modulation of individual m^6^A site in ESCs prevents further exploration to this fundamental scientific question, and hampers the functional characterization of a specific m^6^A modification in cell fate determination.

To investigate the regional effects of m^6^A modification without changing the primary sequence, two recent studies have achieved programmable m^6^A editing by coupling m^6^A demethylases with a RNA‐targeting, catalytically inactive Cas9 (dCas9).^[^ ^16^, ^17^ ^]^ However, this system requires transient transfection of the synthetic PAM‐presenting DNA oligonucleotide (PAMmer) that has a short intracellular half‐life (2–3 days); in addition, the timing of editing cannot be precisely controlled. Thus, it is not suitable for cell fate regulation studies in ESCs which usually take much longer time and require accurate temporal control. The CRISPR‐associated nuclease Cas13 has been shown to target single‐strand RNA (ssRNA) guided by a CRISPR RNA (crRNA) without the need of an artificially synthetic PAMmer.^[^ ^18^, ^19^ ^]^ Similar to dCas9, the catalytically inactivated Cas13 (dCas13) can no longer cleave ssRNA but retains high RNA‐binding affinity.^[^ ^20^, ^21^ ^]^ We speculated that coupling of inducible dCas13 to the m^6^A eraser would be a feasible strategy to establish a targeted RNA m^6^A erasure (TRME) system in human ESCs (hESCs) that allows programmable demethylation of m^6^A at sites specified by a Cas13 crRNA. To test this idea, we attempted to engineer the m^6^A demethylase ALKBH5^[^ ^4^ ^]^ into a programmable RNA base editor by taking advantage of RNA‐guided dCas13a. To weaken nonspecific RNA‐binding affinity of full‐length ALKBH5 and minimize the size of the TRME construct, we only kept the catalytic domain of ALKBH5 (residues 66‐292, referred to as ALK hereafter) which was previously reported to show no catalytic defects.^[^ ^22^ ^]^ We next tethered ALK to either the C‐terminal or N‐terminal of GFP‐tagged dCas13a from *Leptotrichia wadei* ^[^ ^20^ ^]^ via a short flexible linker^[^ ^21^ ^]^ to create dCas13a‐ALK or ALK‐dCas13a (**Figure** **1**a). We found that dCas13a‐ALK was more stable and soluble when recombinantly produced in *E. coli* (Figure S1a, Supporting Information), and therefore proceeded with it in the following experiment. As mammalian m^6^A readers exist within both the cytoplasm and the nucleus, dCas13a‐ALK localized to either part of the cell may access different RNAs and result in distinct biological consequences. We therefore engineered cytoplasm‐ and nucleus‐localized TRME construct variants by adding nuclear export signal (NES) or nuclear localization signal (NLS) sequences to both 5’ and 3’ terminals of each construct, generating dCas13a‐ALKnes (Figure 1b) or dCas13a‐ALKnls (Figure 1c), respectively. An inactive control (dCas13a‐dALK) was created by introducing a H204A mutation to ALK, which was shown to completely abolish the catalytic activity of ALKBH5.^[^ ^4^ ^]^ dCas13a without fusing with ALK, served as another negative control.

**Figure 1:**
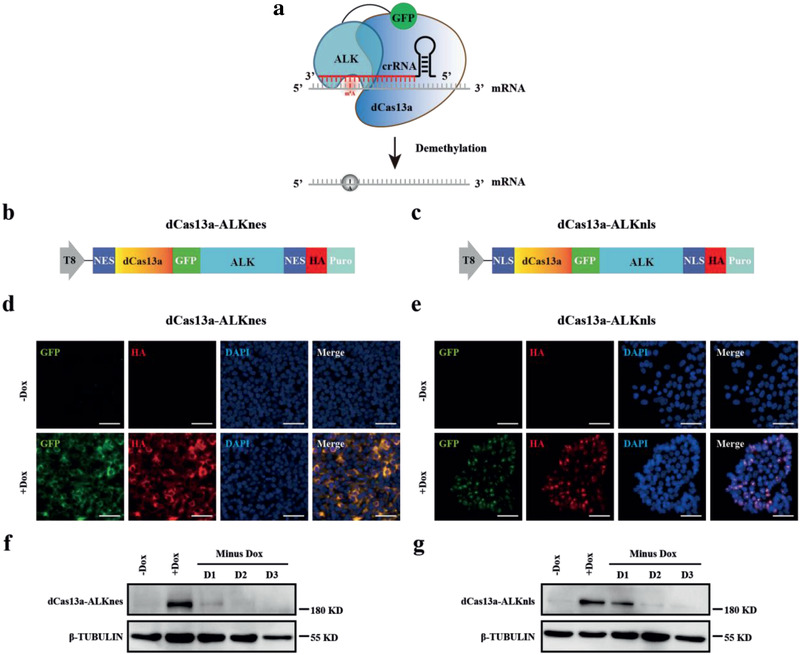
Generation of the inducible TRME hESCs. a) Strategy overview of the TRME system. A programmable RNA‐binding protein dCas13a, when fused to the catalytic domain of ALKBH5 (ALK), mediates the crRNA‐specified demethylation of m^6^A to A site specifically in a target transcript. b,c) Schematic of the TRME editor constructs. ALK, catalytic domain of ALKBH5; NES, nuclear export signal; NLS, nuclear localization signal; HA, hemagglutinin epitope tag; Puro, puromycin resistance gene. The doxycycline‐inducible Tet‐On promoter (T8) drives the transcription of the TRME editors. d,e) Immunostaining analyses of GFP and HA tag, both of which indicate the presence of the TRME editor, in dCas13a‐ALKnes (left) and dCas13a‐ALKnls (right) hESCs with or without doxycycline treatment. Scale bars, 50 µm. f,g) Western blot analysis of the dCas13a‐ALK protein in dCas13a‐ALKnes (left) and dCas13a‐ALKnls (right) hESCs. Total protein was extracted from samples and analyzed by western blot with antibodies against HA tag. β‐TUBULIN was used as a loading control. dCas13a‐ALK protein was robustly induced 24 h after doxycycline treatment and degraded rapidly when doxycycline was removed.

To evaluate these candidate TRME constructs in hESCs, we independently derived multiple stable, single cell‐derived clones of dCas13a‐ALKnes/dCas13a‐ALKnls and the inactive controls by using a piggyBac transposon system that is resistant to transgene silencing during differentiation.^[^ ^23^, ^24^ ^]^ dCas13a‐ALK expression was controlled by a Tet‐On promoter thus can be conditionally upregulated at the desired developmental stages. We found that dCas13a‐ALK protein was barely detectable in the absence of doxycycline, but was robustly induced by doxycycline addition in both dCas13a‐ALKnes and dCas13a‐ALKnls hESCs, revealed by immunostaining and western blots analyses of the TRME editors fused with GFP and hemagglutinin epitopes (Figure 1d–g). We further confirmed that NES‐tagged editors localized in the cytoplasm and NLS‐tagged editors localized in the nucleus (Figure 1d,e), suggesting that intracellular localization of dCas13a‐ALK can be reliably controlled with localization tags. After removing doxycycline, dCas13a‐ALK protein rapidly degraded (Figure 1f,g), thus supporting studies in hESCs that rely on precisely temporal regulation and user manipulation.

Next we further characterized the established TRME hESC lines. During long‐term passaging, they retained a stable undifferentiated morphology and uniform expression of key pluripotent marker proteins, including SOX2, OCT4, and NANOG (Figure S1b, Supporting Information). To determine whether dCas13a‐ALK expression alone would affect the biological properties of hESCs, we continuously cultured the cells with doxycycline for 4 days, and observed no cytotoxicity or decrease in proliferation, self‐renewal capacity, or viability in these cells (Figure S1c–e, Supporting Information). Furthermore, the robust dCas13a‐ALK expression remained virtually unchanged after a continuous culture for 6 months (37 passages) (Figure S1f, Supporting Information), providing a stable and reliable system that was not compromised by transgene silencing.

Using the above suite of cytoplasm‐ and nucleus‐localized TRME editors, we sought to induce crRNA guided, site‐specific m^6^A modifications on endogenous transcripts in hESCs. We first chose to target adenine A1398^[^ ^25^ ^]^ within the 3’‐UTR of *SOX2* mRNA because *SOX2* exhibits the highest degree of methylation (82.7% in hESCs) among the pluripotency genes revealed by m^6^A‐level and isoform‐characterization sequencing (m^6^A‐LAIC‐seq).^[^ ^26^ ^]^ m^6^A enrichment at this site was further confirmed by methylated RNA immunoprecipitation sequencing (MeRIP‐seq) (**Figure** **2**a) and MeRIP‐RT‐qPCR (Figure S2a, Supporting Information).

**Figure 2:**
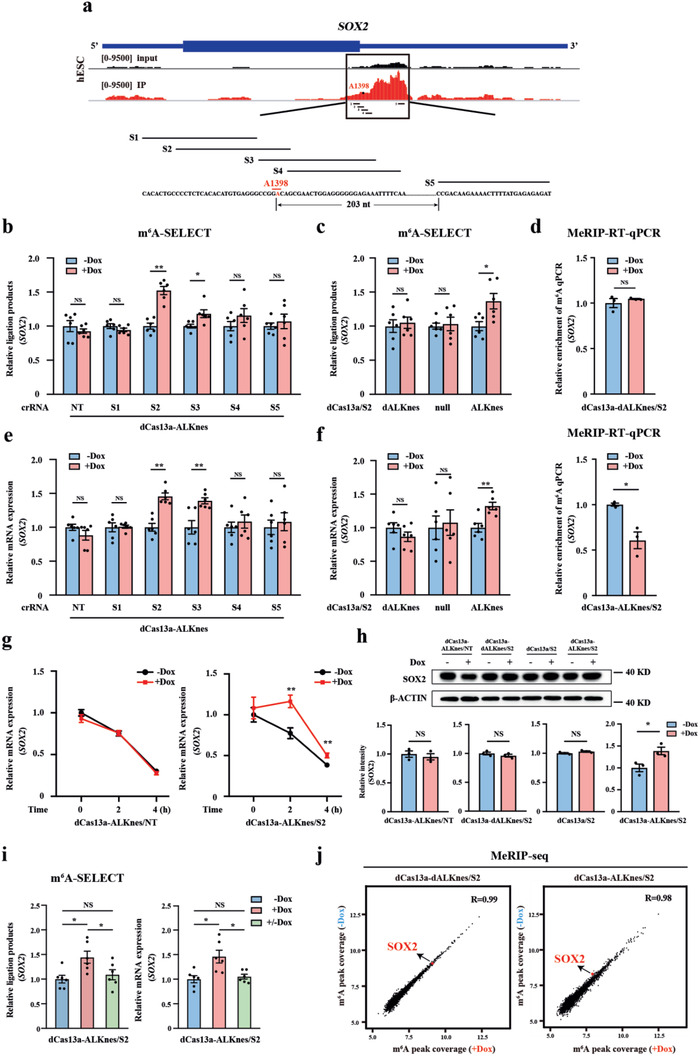
Cytoplasm‐localized TRME editor induces site‐specific m^6^A demethylation and affects target transcript expression in hESCs. a) Distribution of m^6^A peaks across the *SOX2* mRNA transcript. Schematic of *SOX2* targeted site A1398 (red) and the guide crRNA design (black). S1‐5 indicates *SOX2* crRNA1‐5, respectively. b) Measurement of m^6^A levels at A1398 of *SOX2* in dCas13a‐ALKnes hESCs containing either non‐targeting or each *SOX2*‐targeting crRNA with or without doxycycline treatment by SELECT assay. NT indicates non‐targeting crRNA. Dox, doxycycline. *n* = 6 for each group. c) Measurement of m^6^A levels at A1398 of *SOX2* in the catalytically inactive control (dCas13a‐dALKnes/S2), dCas13a alone (dCas13a‐null/S2), or dCas13a‐ALKnes/S2 hESCs with or without doxycycline treatment by SELECT assay. *n* = 6 for each group. d) MeRIP‐RT‐qPCR analysis of m^6^A enrichment on *SOX2* 3’‐UTR in dCas13a‐dALKnes/S2 or dCas13a‐ALKnes/S2 hESCs with or without doxycycline treatment. Primers were designed to span the targeted m^6^A site at A1398 of *SOX2* 3’‐UTR. *n* = 3 for each group. e) RT‐qPCR analysis of *SOX2* mRNA levels in dCas13a‐ALKnes hESCs containing either NT or each *SOX2*‐targeting crRNA with or without doxycycline treatment. *n* = 6 for each group. f) RT‐qPCR analysis of *SOX2* mRNA levels in dCas13a‐dALKnes/S2, dCas13a‐null/S2 or dCas13a‐ALKnes/S2 hESCs with or without doxycycline treatment. *n* = 6 for each group. g) Measurement of *SOX2* mRNA decay in dCas13a‐ALKnes hESCs containing NT or S2 with or without doxycycline treatment. *n* = 6 for each group. h) Representative (up) and quantitative (bottom) results of SOX2 protein expression revealed by western blot analyses in dCas13a‐ALKnes/NT, the catalytically inactive control (dCas13a‐dALKnes/S2), dCas13a alone (dCas13a/S2) or dCas13a‐ALKnes/S2 hESCs with or without doxycycline treatment. β‐ACTIN was used as a loading control. *n* = 3 for each group. i) SELECT (left) and RT‐qPCR (right) analyses of m^6^A levels at A1398 and *SOX2* mRNA levels in dCas13a‐ALKnes/S2 hESCs with continuous doxycycline treatment or after doxycycline withdrawal. +/−Dox indicates doxycycline withdrawal for 24 h. *n* = 6 for each group. NS, not significant (*p* > 0.05), *p < 0.05 using one‐way ANOVA with a post‐hoc Tukey test. j) Scatter plots showing the variation of m^6^A coverage of individual m^6^A peak in dCas13a‐dALKnes/S2 or dCas13a‐ALKnes/S2 hESCs with or without doxycycline treatment. Pair‐wise comparisons were shown between the groups with or without doxycycline treatment in each cell type. The red dot indicates SOX2. The m^6^A coverage was converted by log2. Data are shown as the mean ± S.E.M. NS, not significant (*p* > 0.05), *p < 0.05, **p < 0.01 using unpaired Student's *t*‐test for two‐group comparisons.

To test whether dCas13a‐ALK could decrease the m^6^A signal at A1398, we designed a panel of five guide crRNAs (termed S1 to S5) located at varied distances to A1398 (Figure 2a). A non‐targeting crRNA (NT) was used as the negative control. We introduced these crRNAs into both the dCas13a‐ALK and the inactive control hESCs, selected subclones, and confirmed that crRNA expression was robust and stable during continuous hESC culture by monitoring the mCherry reporter carried in the crRNA constructs (Figure S1f, Supporting Information). We then treated the multiple subclones containing either NT or each *SOX2*‐targeting crRNA with doxycycline and examined the degree of methylation at A1398 using the recently reported SELECT method,^[^ ^27^ ^]^ which enables site‐specific and quantitative m^6^A measurement by elongation and ligation‐based qPCR amplification. With the presence of doxycycline, we observed significant increases of ligated products (an indication of decreased m^6^A levels) only in dCas13a‐ALKnes hESCs harboring S2 or S3, two crRNAs that overlapped with A1398 (Figure 2b). Notably, NT or crRNAs that positioned at only 3 nt (S4) or more (S1 and S5) away from A1398 failed to demethylate the site (Figure 2b), suggesting that the TRME editor has a very accurate editing window and is most active when overlapping with the targeted m^6^A site. This programmable m^6^A erasure was due to the demethylase activity of cytoplasm‐localized TRME editor because neither dCas13a‐dALK nor dCas13a alone altered the m^6^A signal at A1398 when co‐expressed with S2 (Figure 2c). As an independent validation, we conducted MeRIP‐RT‐qPCR and confirmed that S2 could reduce A1398 methylation together with dCas13a‐ALKnes but not the demethylase‐inactive mutant upon doxycycline addition (Figure 2d). Consistently, we also observed an evidently doxycycline‐induced decrease of A1398 methylation in dCas13‐ALKnls hESCs with the presence of S2 or S3 but not other non‐overlapping crRNAs (Figure S2b, Supporting Information).

One major effect of altering m^6^A deposition is to increase or decrease the expression of methylated mRNA through regulating mRNA decay.^[^ ^28^ ^]^ For example, *SOX2* mRNAs had a longer half‐life and could not be properly downregulated with differentiation in METTL3‐deficient hESCs.^[^ ^14^ ^]^ To explore whether the TRME editors characterized above can induce similar changes, we examined the abundance and turnover rate of *SOX2* mRNA with or without doxycycline‐treatment in dCas13a‐ALKnes and dCas13a‐ALKnls hESCs harboring either NT or each *SOX2*‐targeting crRNA. Notably, we observed that dCas13a‐ALKnes but not dCas13a‐ALKnls co‐expressed with S2 or S3 caused substantial increases in *SOX2* mRNA levels (Figure 2e; Figure S2b, Supporting Information), despite the ability of both constructs to significantly decrease m^6^A deposition at A1398 (Figure 2b; Figure S2b, Supporting Information). The lack of RNA abundance changes from m^6^A erasure in dCas13a‐ALKnls hESCs may reflect that m^6^A‐mediated mRNA decay is mediated by the cytoplasm‐localized m^6^A readers YTHDF2. Published YTHDF2 RNA binding protein immunoprecipitation sequencing (RIP‐seq) data^[^ ^29^ ^]^ has confirmed its robust binding with the 3′‐UTR of *SOX2* mRNA in the glioblastoma stem cell line GSC11 (Figure S2c, Supporting Information). By performing YTHDF2 RIP‐RT‐qPCR, we further confirmed the binding between YTHDF2 and *SOX2* mRNA in hESCs, and this interaction was decreased together with the dCas13a‐ALKnes‐induced *SOX2* demethylation (Figure S2d, Supporting Information). Furthermore, NT control or crRNAs that were insufficient to demethylate A1398 did not affect *SOX2* mRNA amounts in dCas13a‐ALKnes hESCs (Figure 2e), indicating the high specificity of TRME editors. As expected, dCas13a alone or catalytically inactive variant failed to phenocopy the effects of dCas13a‐ALKnes even with the presence of S2 guide (Figure 2f), suggesting that the increased expression of *SOX2* upon dCas13a‐ALKnes induction resulted from altered A1398 methylation. This notion was further supported by the observation that S2 but not NT markedly increased *SOX2* mRNA stability in dCas13a‐ALKnes hESCs (Figure 2g). Consistent with the increased stability and abundance of *SOX2* mRNA, the protein level of SOX2 was also evidently upregulated upon doxycycline administration only in the dCas13a‐ALKnes/S2 hESCs but not the negative control cells (Figure 2h).

We further validated these results in additional m^6^A sites, including *METTL14* A1404, *JDP2* A1024, *DUSP5* A1613, which exhibit high degree of methylation in hESCs.^[^ ^26^ ^]^ In agreement with what we have observed at *SOX2*, we found that dCas13a‐ALKnes guided by crRNAs that overlapped with the targeted m^6^A loci increased the amount (Figure S2e, Supporting Information) and stability (Figure S2f, Supporting Information) of corresponding transcripts, whereas crRNAs located at 30–130 nt away from the targeted site did not (Figure S2e, Supporting Information). To further validate these effects were specific to YTHDF2 and m^6^A, we introduced small hairpin RNA for *YTHDF2* into the dCas13a‐ALKnes hESC lines harboring crRNAs that target either *SOX2*, *METTL14*, *JDP2*, or *DUSP5* (Figure S3a, Supporting Information), and examined the expression of these genes with or without doxycycline treatment by RT‐qPCR. Notably, we observed that dCas13a‐ALKnes‐induced increases in mRNA abundance of these genes were abolished with YTHDF2 knockdown (Figure S3b, Supporting Information). These results collectively confirmed that cytoplasm‐restricted TRME editor is able to achieve site‐specific m^6^A modification and affect target transcript expression through regulating m^6^A‐mediated mRNA degradation.

Next, we explored the consequence of nuclei‐restricted m^6^A erasure induced by dCas13a‐ALKnls. In the nucleus, m^6^A modification is known to affect mRNA transport to the cytoplasm.^[^ ^30^ ^]^ We thus examined the effect of dCas13a‐ALKnls‐induced demethylation on nuclei‐to‐cytoplasm transport of the nascent *SOX2* mRNA. We separated the cytoplasm and nucleus of dCas13a‐ALKnls/S2 cells to examine the change in nucleus to cytoplasm ratio (NCR) of *SOX2* with or without doxycycline treatment. Interestingly, we observed a dramatic increased *SOX2* NCR upon doxycycline administration (Figure S4a, Supporting Information), indicating that site‐specific m^6^A demethylation in the nucleus may hamper nuclei‐to‐cytoplasm transport of the transcribed mRNA. This notion was further supported by the fact that the protein expression of *SOX2* was also downregulated by the dCas13a‐ALKnls editor (Figure S4b, Supporting Information), in contrast to cytoplasm‐restricted m^6^A demethylation which adversely increased the expression of SOX2.

We next sought to determine if demethylation induced by TRME editor could be reversed by removing doxycycline from the cell culture. We found that adding doxycycline for 48 h in S2‐containing dCas13a‐ALKnes hESCs increased the SELECT ligated products by 44.6% and *SOX2* mRNA abundance by 45.9%, respectively, which were completely restored after doxycycline withdrawal for only 24 h (Figure 2i). These findings indicate that TRME‐induced programmable m^6^A editing and altered gene expression are fully reversible in hESCs.

To access whether crRNA‐guided TRME editor would induce substantial nonspecific demethylation, we first examined the effect of dCas13a‐ALKnes on the total m^6^A content within hESCs by capturing cellular mRNA and staining with anti‐m^6^A antibodies. Similar to the demethylase‐inactive control, ectopic expression of dCas13a‐ALKnes by doxycycline administration produced no significant difference in global m^6^A abundance within hESCs harboring the *SOX2* S2 guide (Figure S5a, Supporting Information). To further test whether crRNA‐guided TRME editor affects the distribution of transcriptome‐wide RNA methylation, we conducted MeRIP‐seq and compared the entire m^6^A methylome in S2‐containing dCas13a‐ALKnes hESCs with or without doxycycline‐treatment. Once again, the inactive dCas13a‐dALKnes hESCs were used as a negative control. We found that the overall m^6^A landscapes across the transcriptome were comparable in these cells (Figure S5b, Supporting Information). Pair‐wise comparison of individual m^6^A peak in dCas13a‐ALKnes hESCs with or without doxycycline‐treatment showed modest variation, no larger than the demethylase‐inactive control (Figure 2j). As expected, doxycycline‐treatment failed to decrease the *SOX2* m^6^A peak abundance in the demethylase‐inactive control cells (Figure 2j, left). In contrast, we observed a 20.3% decrease in the *SOX2* m^6^A peak intensity in the dCas13a‐ALKnes/S2 hESCs upon doxycycline addition (Figure 2j, right), indicating a demethylation degree that is roughly half as strong as what was detected by the SELECT method (44.6%) which only measured a single site, A1398. We consider it a reasonable observation because two adjacent m^6^A sites (A1398 and A1405) locate in this peak, thus the doxycycline‐altered m^6^A peak intensity detected by MeRIP‐seq may contribute by both two sites, with only A1398 actually demethylated by TRME editing. This further supports the notion that TRME has a very accurate editing window and great specificity for single site modulation.

Besides *SOX2*, we observed 49 other loci among a total of 2643 peaks showed differential modification intensity between the Dox^+^ and Dox^−^ dCas13a‐ALKnes/S2 samples (>20% decrease in the m^6^A peak intensity). However, off‐target prediction^[^ ^31^ ^]^ revealed that none of these loci was derived from crRNA‐guided demethylation. Even using very loose base‐pairing criteria (8 mismatches), only one locus emerged as a potential off‐target site (Table S1, Supporting Information). These results collectively suggest that the possible off‐target activity of cytoplasm TRME editor is either modest or beyond the detection threshold.

Next, we determined whether site‐specific m^6^A modulation would be sufficient to affect cell fate determination of hESCs. Since *SOX2* is a known hESC master gene and is highly m^6^A modified, we evaluated the functional consequence of *SOX2* A1398 demethylation on hESC differentiation by utilizing the TRME system characterized above. dCas13a‐ALKnes/S2 hESCs could be stably maintained in undifferentiated culture condition after doxycycline induction, suggesting that A1398 demethylation did not affect hESC self‐renewal or viability. We then targeted differentiated dCas13a‐ALKnes/S2 hESCs toward either endoderm, mesoderm, or ectoderm, respectively. Strikingly, upon induction of differentiation, doxycycline‐treatment prolonged SOX2 expression in dCas13a‐ALKnes/S2 hESCs but not the demethylase‐inactive control cells (**Figure** **3**a–c; Figure S6, Supporting Information). Meanwhile, the expression of several key endodermal and mesodermal genes was significantly downregulated, whereas genes important for ectoderm formation were evidently upregulated upon doxycycline administration only in dCas13a‐ALKnes/S2 hESCs (Figure 3a–c), suggesting that A1398 demethylation promotes ectodermal but inhibits endodermal and mesodermal specification of hESCs. This observation is consistent with several previous studies in which prolonged SOX2 expression has been shown to enhance ectodermal differentiation but impede mesodermal/endodermal specification of ESCs.^[^ ^32^, ^33^, ^34^ ^]^ This conclusion was further validated by immunofluorescence staining analyses of key germ‐layer marker proteins, where doxycycline‐treatment significantly decreased the percentage of SOX17^+^/FOAX2^+^ endodermal cells and BRACHYURY^+^ mesodermal cells, and increased the ratio of PAX6^+^/SOX2^+^ ectodermal cells in a manner dependent on an A1398‐targeting S2 guide, active demethylase and their fusion to cytoplasm‐restricted dCas13a (Figure 3d–f). In aggregate, these findings establish that temporal erasure of a single m^6^A site is sufficient to produce distinct lineage choice outcomes in hESCs, further highlighting the importance of m^6^A‐dependent epitranscriptional control in cell fate transitions. m^6^A modification on master genes serves as a timely maintainer of the balance between pluripotency and lineage priming factors, and may work as a “plug‐in” to other pre‐existing pathways by altering downstream gene expression. In this manner, m^6^A modifications can promote a fast response to external cues during times of cell fate transition.

**Figure 3:**
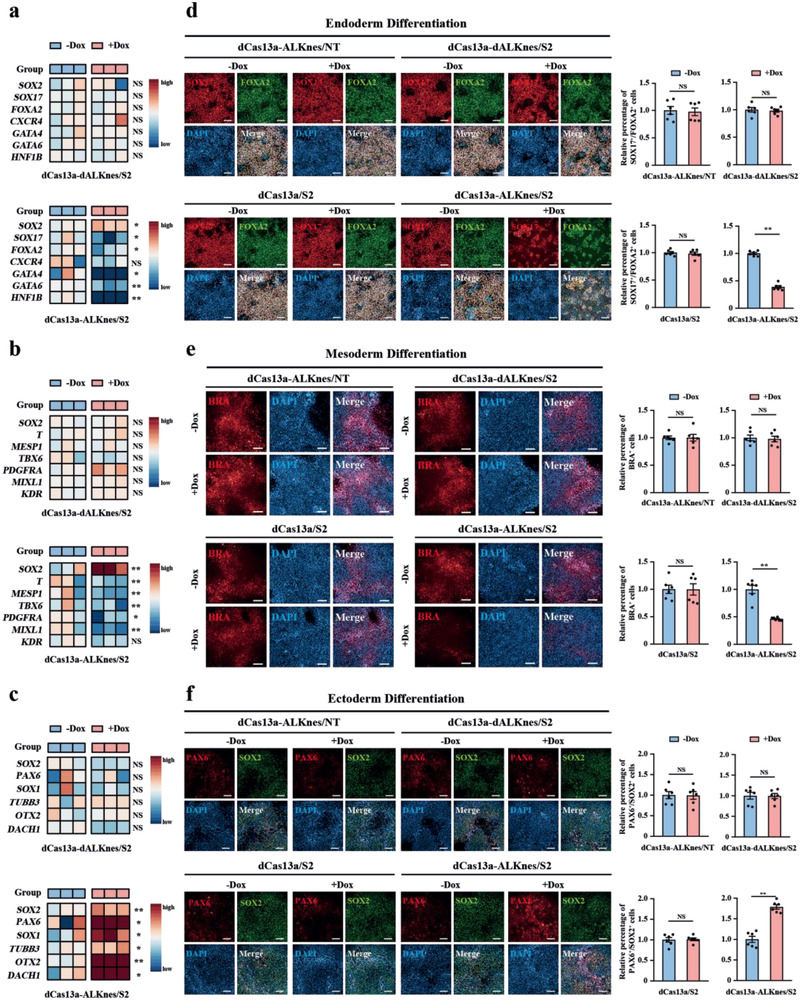
Site‐specific demethylation of *SOX2* affects germ‐layer commitment of hESCs. a–c) Heatmap showing mRNA expression of *SOX2* and a) endoderm‐, b) mesoderm‐, or c) ectoderm‐marker genes in dCas13a‐dALKnes/S2 or dCas13a‐ALKnes/S2 hESCs that underwent either endoderm (3 days), mesoderm (1.5 days), or ectoderm (4 days) differentiation, respectively, in the presence or absence of doxycycline. *n* = 3 for each group. NS, not significant (*p* > 0.05), *p < 0.05, **p < 0.01 using unpaired Student's *t*‐test. d–f) Immunofluorescence analyses of d) endoderm markers SOX17/FOXA2, e) mesoderm marker BRACHYURY (BRA), or f) ectoderm markers PAX6/SOX2 in dCas13a‐ALKnes/S2 and other negative control hESCs that underwent either endoderm (3 days), mesoderm (1.5 days), or ectoderm (4 days) differentiation, respectively, in the presence or absence of doxycycline. Scale bars, 100 µm. *n* = 6 for each group. Data are shown as the mean ± S.E.M. NS, not significant (*p* > 0.05), **p < 0.01 using unpaired Student's *t*‐test.

In sum, by coupling the RNA‐targeting capability of CRISPR‐dCas13 with m^6^A eraser, we established a stable system to achieve rapid, adjustable, and site‐specific demethylation of RNAs in hESCs. It offers researchers a versatile toolbox to understand the regional effects of mRNA methylation in hESCs, enabling studies to unlock the secrets of cell fate control at the emerging new tunable layer termed epitranscriptome. Application of this system leads to the proof‐of‐concept demonstration that demethylation of a single m^6^A site rather than global m^6^A remodeling is sufficient to affect stem cells to adopt new cell fates.

The TRME system described in the present study possesses many unique advantages from a stem cell biology perspective. First, by combining a doxycycline‐inducible construct and a piggyBac transposon‐based transgene delivery strategy, the TRME hESC lines developed here are resistant to transgene silencing during expansion and differentiation, and allow for controllable and reversible modulation of a chosen m^6^A site upon addition or removal of doxycycline. This system helps us precisely control the timing of editing within a timeframe long enough for cells to adopt a new fate, thus it is useful in hESC differentiation studies that are usually highly dynamic and time‐consuming. Second, the TRME system does not require any laboratory‐synthesized components that are incompatible to stable transfection, such as the modified PAMmer oligonucleotides used by the dCas9‐directed m^6^A editor.^[^ ^16^ ^]^ TRME thus can be genetically encoded in their entirety by creating stable hESC clones carrying the crRNA of interest in the genome. This strength enables the generation of cell libraries for genome‐scale screen of functional m^6^A sites important for cell fate determination in hESCs, a strategy proved to be successful in other CRISPR systems.^[^ ^35^ ^]^ Third, the TRME editor designed in this study has a very small editing window (≈30 nt) and is most active only when directly overlaps with the targeted m^6^A loci, thus conferring greater specificity for single site modulation. This advantage distinguishes the TRME system developed here from the recently reported dm^6^ACRISPR editor, which uses full‐length ALKBH5 fusion to dCas13b.^[^ ^36^ ^]^ This dm^6^ACRISPR system, unlike TRME editor, has a much larger editing window and can demethylate m^6^A sites up to 3050 nt away from the guide, thus synergistically affected multiple adjacent adenines susceptible to methylation by a single crRNA transfection.^[^ ^36^ ^]^ This difference may result from the fact that TRME contains only a catalytic domain of ALKBH5, thus has less nonspecific RNA‐binding affinity when compared with using full‐length ALKBH5.

In light of these strengths, the TRME system described here provides a powerful platform to dissect the causal relationships between the presence of a specific m^6^A and lineage decision outcomes in hESCs. By coupling with a genome‐wide genetic screen approach, we anticipate the TRME hESC lines as suitable model systems to systematically identify factors that control cell fates via epitranscriptional mechanisms. Given the broad applicability of the strategy and the versatility of CRISPR toolkits on the rise, our TRME approach may be developed to enable reversible epitranscriptional editing in many other biological systems for similar purposes, or in disease contexts where hypermethylation of a chosen transcript is pathogenic.

## Experimental Section

The experimental procedures and materials used are included in the Supporting Information.

## Conflict of Interest

The authors declare no conflict of interest.

## Supporting information

Supporting Information

## Data Availability

The data that support the findings of this study are available from the corresponding author upon reasonable request.
