# Supplementary material for: Targeted RNA N 6‐Methyladenosine Demethylation Controls Cell Fate Transition in Human Pluripotent Stem Cells
Source: Adv Sci (Weinh). 2021 Mar 18;8(11):2003902. doi: 10.1002/advs.202003902 (PMC8188216; doi:10.1002/advs.202003902)
Supplement: Supplementary file 1 — Supporting Information [file ADVS-8-2003902-s001.pdf]

© 2021 Wiley-VCH GmbH

ADVANCED SCIENCE
Open Access

Supporting Information

for *Adv. Sci.,* DOI: 10.1002/advs.202003902

Targeted RNA N_6-Methyladenosine Demethylation Controls Cell Fate Transition in Human Pluripotent Stem Cells

*Xuena Chen, Qingquan Zhao, Yu-Li Zhao, Guo-Shi Chai, Weisheng Cheng, Zhiju Zhao, Jia Wang,*Guan-Zheng Luo,*and Nan Cao**

Supporting Information

Targeted RNA *N*^{6}-Methyladenosine Demethylation Controls Cell Fate Transition in Human Pluripotent Stem Cells

Xuena Chen, Qingquan Zhao, Yu-Li Zhao, Guo-Shi Chai, Weisheng Cheng, Zhiju Zhao, Jia Wang, Guan-Zheng Luo,* and Nan Cao**

Experimental Methods

Plasmid construct design. Full-length coding sequences (CDSs) of human ALKBH5 were commercially synthesized and cloned into pUC57 vector (Sangon Biotech) to generate pUC57-ALKBH5 plasmid. DNA sequences encoding ALK, the catalytic domain of ALKBH5 (residue 66-292), were amplified from the pUC57-ALKBH5 plasmid using 2 × Phanta Max Master Mix (Vazyme). dCas13a CDSs were amplified from pC015-dLwCas13a-NF vector (Addgene plasmid no.91905). TRME editors were constructed by fusing ALK to the N- or C-terminus of dCas13a via a short flexible linker (GSGGGGGS). For protein expression in *E. coli*, TRME constructs were subcloned into the pET-28b vector. For programmable RNA demethylation in hESCs, TRME constructs were subcloned into the Tet-On inducible piggyBac transposon plasmid using ClonExpress® MultiS One Step Cloning Kit (Vazyme). Cytoplasm- and nucleus-localized editors introduced nuclear export signals (LPPLERLTL) and nuclear localization signals (KRPAATKKAGQAKKKK) at both 5' and 3' terminals of each construct, respectively. The demethylase-inactive control (dCas13a-dALK) was generated by installing a H204A mutation to ALK using Mut Express MultiS Fast Mutagenesis Kit V2 (Vazyme). crRNAs contained 28-nt spacers flanked by a 36-nt direct repeat (DR). The sequence of DR is GAUUUAGACUACCCCAAAAACGAAGGGGACUAAAAC. Spacers were designed by using the CRISPR-RT tool (http://bioinfolab.miamioh.edu/CRISPR-RT/index.php). For efficient m^6A targeting, the number of bases between the 3' end of the spacer and the targeted m^6A site was restricted to 3 or less and crRNA that overlapped with the targeted m^6A site was preferred. Then the crRNAs were subjected to MEGABLAST (https://blast.ncbi.nlm.nih.gov/Blast.cgi) for further validation to avoid potential mismatching to other human mRNAs. The full-length DR together with the U6 promoter was amplified from the pC016-LwCas13a plasmid backbone (Addgene plasmid no. 91906) and golden-gate cloned into pSLQ1371 to produce the crRNA expression plasmid, which has constitutive guide RNA expression under a U6 promoter. Then the designed 28-nt spacers were golden-gate cloned into the crRNA expression plasmid using ClonExpress® MultiS One Step Cloning Kit (Vazyme). shRNA oligos targeting *YTHDF2* were annealed and cloned into the lentiviral vector pLKO.1-BSD. Sequences of crRNAs or shRNA used in this study are listed as followed (sequences are from 5' to 3'):

SOX2-crRNA1(S1): CCCUCACAUGUGUGAGAGGGGCAGUGUG;

SOX2-crRNA2(S2): CUGUCCGGCCCUCACAUGUGUGAGAGGG;

SOX2-crRNA3(S3): UUUCUCCCCCCUCCAGUUCGCUGUCCGG;
SOX2-crRNA4(S4): UUGAAAAUUUCUCCCCCCUCCAGUUCGC;
SOX2-crRNA5(S5): AUCUCUCUCAUAAAAGUUUUCUUGUCGG;
JDP2-crRNA1(J1): CAGUUUCAUUCUGAGUUCCAUCCUCAGC;
JDP2-crRNA2(J2): CUGUCCUGCCUCCUUCAAGUGGGGCCCC;
DUSP5-crRNA1(D1): CAGUCCCGAGAACCUACCCUGAGGUCCG;
DUSP5-crRNA2(D2): GGGUCUGGAAGUACAGUAGUCAGCACAU;
METTL14-crRNA1(M1): AUGUCUUCAACAAUUAUCGAGGUGGAAA;
METTL14-crRNA2(M2): AAGUUCCACCUCUUCCUCCACCUCUGGG;
Non-targeting crRNA(NT): UAGAUUGCUGUUCUACCAAGUAAUCCAU;
YTHDF2-shRNA: GATGGATTAAACGATGATGAT.

Bacterial protein expression. dCas13a-ALK or ALK-dCas13a constructs were subcloned into the pET-28b vector and verified by DNA sequencing. *E. coli* strain BL21 (DE3) that received the recombinant plasmids transfection were grown in LB medium at 37 °C until A600 reached 0.8-1.0, and protein expression was induced overnight at 16 °C by addition 1.0 mM Isopropyl β-D-Thiogalactoside (IPTG). Lysed samples were centrifuged (12,000 g for 2 min at 4 °C) and the supernatant was collected. Protein concentration was quantified with the BCA Protein Assay Kit (Beyotime) followed by heating for 15 min at 95 °C. Boiled samples were harvested by centrifuging at 4,000 g for 10 min, loaded on SDS-PAGE gels and separated by electrophoresis for about 2 hours. Gels were then removed from the plates and visualized by coomassie blue staining.

Mammalian cell culture. HEK293T cells were cultured in high-glucose Dulbecco's Modified Eagle Medium (Hyclone) supplemented with 10% fetal bovine serum (Hyclone). NKX2-5$^{eGFP/w}$ hESCs were maintained in E8 medium (STEMCELL Technologies) on Matrigel-coated (BD Biosciences) plates with daily medium changes. At about 60%-70% confluency, NKX2-5$^{eGFP/w}$ hESCs were washed with phosphate-buffered saline (PBS, Hyclone) once and incubated in 0.5 mM EDTA (Thermo Fisher Scientific) for 3-5 minutes at room temperature. EDTA was removed from the plate and the cells were gently washed off via E8 medium containing Rho-associated protein kinase (Rock) inhibitor Y-27632 (5 μM, Selleck)[1] and passaged onto Matrigel-coated plates at 1:6 to 1:8 dilutions. Rock inhibitor was removed on the next day after passage. Both cells were incubated at 37 °C with 5% CO_2.

Lentivirus production. HEK293T cells were seeded in 10 cm dishes. At about 90% confluency, cells were transfected with 16 μg lentiviral vector, 4 μg pMD2.G and 12.5 μg psPAX2 using BES buffered saline (Sigma). The lentivirus supernatant was harvested at 48 and 72 hours after transfection, mixed together, centrifuged at 600 g for 10 min, passed through a 0.45 μm filter and stored at -80 °C.

Construction of the TRME hESC lines. NKX2-5$^{eGFP/w}$ hESCs were enzymatically dissociated into single cells by Accutase (STEMCELL Technologies) and nucleofected with TRME editor plasmid (or the corresponding negative control plasmids) and transposase plasmid at a mass ratio of 1,000:1 using the Neon® Transfection System (Thermo Fisher Scientific). Then cells were seeded onto 6-well plates in E8 medium supplemented with Y-27632 (5 μM). After nucleofection, puromycin (1 μg ml^{-1}, Selleck) and doxycycline (1 μg ml^{-1}, Sigma) were added for transfected cell selection until stable colonies appeared. Fluorescence-activated cell sorting (FACS) was carried out to isolate GFP positive cells which were then seeded onto 6-well plate at a density of 2×10^5 cells per well and infected with crRNA-expressing lentiviruses at 30% confluency for 6 hours. After two rounds of transduction, TRME hESCs expressing both GFP and mCherry were sorted by FACS and expanded in following cell culture.

Differentiation of TRME hESCs into endoderm, mesoderm and ectoderm. For *in vitro* differentiation assays, TRME hESCs were enzymatically dissociated into single cells by Accutase and seeded onto Matrigel-coated dishes in E8 medium supplemented with Y-27632 (5 μM). When grown to 80%-90% confluency 2-3 days after plating, culture medium was switched to differentiation medium and referred to as day 0. For endoderm differentiation, the medium was changed to E8 basal medium (DMEM/F-12 (Thermo Fisher Scientific) containing L-ascorbic acid (64 μg ml^{-1}, Sigma), sodium selenite (14 ng ml^{-1}, Sigma), transferrin (10.7 μg ml^{-1}, Sigma) and 1 × Chemically Defined Lipid Concentrate (Thermo Fisher Scientific)) supplemented with Activin A (10 ng ml^{-1}, PeproTech) from day 0 to day 3. CHIR99021 (3 μM, Selleck) was also added at day 0 for 24 hours and removed thereafter. For mesoderm differentiation, the medium was changed from E8 to E8 basal medium supplemented with FGF2 (20 ng ml^{-1}, R&D), LY294002 (10 μM, Sigma), and BMP4 (10 ng ml^{-1}, R&D) for 36 hours. For ectoderm differentiation, the medium was changed from E8 to E6 medium (DMEM/F-12 containing L-ascorbic acid (64 μg ml^{-1}), sodium selenite (14 ng ml^{-1}), transferrin (10.7 μg ml^{-1}) and insulin (19.4 μg ml^{-1}, Sigma)) and the cells were cultured in this condition for 4 days before further analyses.

Immunofluorescence staining. TRME hESCs were washed with PBS and fixed by 4% paraformaldehyde (Meilunbio) for 15 min at room temperature. The fixed cells were then washed three times with PBS and treated with 0.3% Triton X-100 (Sigma) at room temperature for 30 min for permeabilization followed by incubation with 3% BSA (Solarbio) for 1 hour. After blocking, cells were incubated with primary antibodies overnight at 4 ºC and then stained with isotype-matched Alexa Fluorescence-conjugated secondary antibodies (Thermo Fisher Scientific) for 1 hour at room temperature. DAPI (1:1,000 dilution)-stained compartments serve as markers of the nuclei. Image acquisition was performed on Operetta CLS and processed with Harmony 4.5 software. Primary antibodies are listed as followed: HA-Tag (51064-2-AP, Proteintech; 1:500); mCherry (GTX128508, GeneTex; 1:300); SOX17 (AF-1924, R&D; 1:500); FOXA2 (8186S, CST; 1:500); SOX2 (AB5603, Millipore; 1:500); OCT4 (SC-5279, Santa Cruz Biotechnology; 1:500); NANOG (3580S, CST; 1:500); BRACHYURY (AF-2085, R&D; 1:200); PAX6 (MA1-109, Thermo Fisher Scientific; 1:200).

Western blot. Cells were digested by 0.5 mM EDTA and washed twice in PBS. Then cells were pelleted, and re-suspended in RIPA buffer (CST) containing the protease inhibitor cocktail (MCE) for 30 min on ice. Lysed samples were centrifuged (12,000 g for 2 min at 4 °C) and supernatant collected. Protein concentration was quantified with BCA Protein Assay Kit (Beyotime) followed by heating for 15 min at 95 °C. Boiled samples were loaded on SDS-PAGE gels and separated by electrophoresis for about 2 hours. Gels were then removed from plates and transferred onto the Immobilon-P membranes (Millipore) and the polyvinylidene difluoride membranes were blocked in TBST buffer containing 5% skimmed milk (BD Biosciences) for 1 hour. Subsequently, the membranes were incubated with primary antibodies overnight at 4 °C followed by blotting with secondary antibodies at room temperature for 2 hours. Blotting signal was visualized after reaction with enhanced chemiluminescence (ECL Plus, GE Healthcare). Primary antibodies are listed as followed: HA (66006-1-Ig, Proteintech; 1: 1,000); β-TUBULIN (AF1216, Beyotime; 1: 1,000); SOX2 (AB5603, Millipore; 1:1,000); β-ACTIN (AF0003, Beyotime; 1: 1,000).

Alkaline phosphatase (AP) staining. AP staining was performed using the Alkaline Phosphatase Detection Kit (Sigma-Aldrich) following the manufacturer's instruction.

Cell viability assays. Cell viability was analyzed by simultaneous fluorescence staining of viable and dead cells using Calcein-AM/PI Double Staining Kit (DOJINDO) and 8.1 μM Hoechst (Thermo Fisher Scientific). Incubation was performed for 15 min in PBS at 37°C and

5% CO_2. Calcein-AM emitted green fluorescence signal in viable cells. Conversely, PI reached nuclei of dead cells only where it emitted red fluorescence.

Cell proliferation assays. For the growth curve assay, cells were seeded on 96-well plates at a density of 2 × 10^4 cells per well. After the indicated incubation time, CCK8 (GLPBIO) was added to each well and further incubated for 2 hours at 37 °C. OD450 absorbance was measured with a microplate reader to detect the number of cells.

SELECT for detection of m^{6}A. SELECT qPCR was conducted by following Xiao's protocol.[2] Briefly, total RNA was isolated from PBS-washed cells using FastPure Cell/Tissue Total RNA Isolation Kit (Vazyme) according to the manufacturer's guide. 2 μg total RNA was incubated with 40 nM up Primer, 40 nM down Primer and 5 μM dNTP in 17 μl 1 × CutSmart buffer (NEB) and annealed in the programs below: 90 °C (1 min), 80 °C (1 min), 70 °C (1 min), 60 °C (1 min), 50 °C (1 min) and 40 °C (6 min). Subsequently, the 17 μl annealing products were incubated with 3 μl enzyme mixtures containing 0.01 U Bst 2.0 DNA polymerase (NEB), 0.5 U SplintR ligase (NEB) and 10 nmol ATP (NEB). The final 20 μl reaction mixtures were incubated at 40 °C for 20 min, denatured at 80 °C for 20 min and kept at 4 °C. SELECT qPCR was performed in a LightCycler 480 Real-Time PCR System (Roche Applied Science) with the following program: 95 °C (5 min), 95 °C (10 s), 60 °C (35 s) for 40 cycles, 95 °C (15 s), 60 °C (1 min), 95 °C (15 s) and 4 °C (hold). The SELECT products of indicated site were normalized to the RNA abundance of indicated transcript bearing this site. Primers are listed as followed (U, up; D, down; sequences are from 5' to 3'):
non-m^{6}A-SELECT-*SOX2*-U:
TAGCCAGTACCGTAGTGCGTGTCACATGTGTGAGAGGGGCAG;
non-m^{6}A-SELECT-*SOX2*-D:
GTGCCGTTAATGGCCGTGCCCAGAGGCTGAGTCGCTGCAT;
m^{6}A-SELECT-*SOX2*-U:
TAGCCAGTACCGTAGTGCGTGTCTCCCCCCTCCAGTTCGCTG;
m^{6}A-SELECT-*SOX2*-D:
CCGGCCCTCACATGTGTGAGACAGAGGCTGAGTCGCTGCAT.

RNA isolation and purification. Cells were washed with PBS and lysed with TRIzol (Thermo Fisher Scientific). Then trichloromethane (Guangzhou Chemical Reagent Factory) was used to extract the total RNA from the TRIzol lysate. Isopropanol (Sangon Biotech) was used to

precipitate RNA from the mixture. mRNA was purified from the total RNA by using the Dynabeads™ mRNA Purification Kit (Thermo Fisher Scientific).

RNA binding protein immunoprecipitation (RIP). Cells were harvested in 1 x polysome lysis buffer (10 mM HEPES, pH 7.0; 100 mM KCl; 5 mM $MgCl_2$; 0.5 % NP-40; 1 mM DTT; 100 U ml^{-1} RRI; 1 × cocktail; 400 μM RVC). Cell lysates were incubated on ice for 15 min, frozen by liquid nitrogen, and stored at -80 °C. Thawed cell lysates were centrifuged at 4 °C, 15,000 g for 10 min. Supernatants were incubated with 10 μl protein G magnetic beads (Thermo Fisher Scientific) with orbital rotation at 4 °C for 45 min. Protein G beads were discarded and the remaining supernatant were then incubated with 10 μl anti-YTHDF2 antibody (Proteintech Group) in 750 μl NT-2 buffer (50 mM Tris-HCl, pH7.4; 1 mM $MgCl_2$; 150 mM NaCl; 0.05 % NP-40), and rotated overnight at 4 °C. After two washes in the NT-2 buffer, the RNP-antibody-protein G mixture was incubated with 100 μl NT-2 buffer plus 0.3 μg μl^{-1} Proteinase K (Sangon Biotech) at 55 °C for 5 min. The input and IP RNA samples were purified with TRIzol and used for RT-qPCR.

m^6A immunoprecipitation. mRNA was fragmented by using the RNA Fragmentation Kit (Thermo Fisher Scientific) at 94 °C for 30 s and enriched by alcohol precipitation. A portion of fragmented RNA was saved as input. Remaining fragmented RNA was subjected to m^6A immunoprecipitation. 25 μl protein G magnetic beads (Thermo Fisher Scientific) were washed three times by IP reaction buffer (150 mM NaCl; 10 mM Tris-HCl, pH 7.4; 0.1% Igepal CA-630 in nuclease-free H_2O), re-suspended in 250 μl reaction buffer, and tumbled with 2 μl anti-m^6A antibody (NEB) at 4 °C for 1 hour. After two washes in reaction buffer, 1 μg fragmented RNA was added to the re-suspended antibody-bead mixture. Then, mRNA-antibody-bead mixture was incubated with orbital rotation at 4 °C for 3 hours. To remove unbound RNA, samples were washed by each of the following buffers: reaction buffer (150 mM NaCl; 10 mM Tris-HCl, pH 7.4; 0.1% Igepal CA-630 in nuclease-free H_2O), low-salt reaction buffer (50 mM NaCl; 10 mM Tris-HCl, pH 7.4; 0.1% Igepal CA-630 in nuclease-free H_2O) and high-salt reaction buffer (500 mM NaCl; 10 mM Tris-HCl, pH 7.4; 0.1% Igepal CA-630 in nuclease-free H_2O). m^6A immunoprecipitation products were eluted with 130 μl elution buffer (150 mM NaCl; 10 mM Tris-HCl, pH 7.4; 0.1% Igepal CA-630; 6.7 mM m^6A in nuclease-free H_2O) at 37 °C, 1,000 rpm for 1.5 hours and purified by alcohol precipitation. Eluted RNA was used for further RT-qPCR.

MeRIP-seq. The m^6A immunoprecipitation method was the same as above. Input samples and IP samples were applied for NGS library construction by using the NEBNext® Ultra II Directional RNA Library Prep (NEB) according to the manufacturer's guide. The adaptor ligated DNAs amplified by PCR for 15 cycles were sequenced on the HiSeqX sequencing platform. MeRIP-seq data were deposited in the NCBI's Gene Expression Omnibus (GEO) database and can be accessed by the accession number GSE158421.

Analysis of MeRIP-seq data. Quality control was performed on raw data with FastQC (www.bioinformatics.babraham.ac.uk/projects/fastqc/). Cutadapt (pypi.org/project/cutadapt/) was used to find and remove adapter sequences. Clean data was mapped to human reference genome (hg19) using HISAT2[3] with default parameters. Multiple-alignment reads were filtered using Samtools to retain reads with map quality (MapQ) scores above 30 and with properly oriented read mates. Bam files were converted to bed files using in-house python scripts. The m^6A peaks were identified using MACS2 (-f BEDPE --nomodel --keep-dup all -q 0.01).[4] FPKM value was calculated to represent m^6A peak intensity. The identified m^6A peak regions were further filtered to assess TRME's specificity. In detail, the m^6A peak region intensity in IP sample is at least 20% higher than that in input sample, and m^6A peak intensity in input sample is more than 20. The *SOX2*-crRNA2(S2) (CUGUCCGGCCCUCACAUGUGUGAGAGGG) was scanned across the transcript sequence covered by final 2643 m^6A peaks for searching mismatched loci with Cas-OFFinder[5] allowing less than or equal to 8 mismatches.

Isolation of cytoplasmic and nuclear RNAs. The cytoplasmic and nuclear RNAs fractionation procedure was performed as described previously with some modifications.[6] Briefly, cells were trypsinized and washed once with cold PBS, and then resuspended in 1 volume of HLB+N buffer (10 mM Tris-HCl, pH 7.4; 10 mM NaCl; 2.5 mM $MgCl_2$; 0.5% Igepal CA-630) supplemented with 1 × RNasin Plus RNase Inhibitor (Promega) for 5 min on ice. The homogenate was then slowly added to 4 volumes of HLB+2N3S buffer (10 mM Tris-HCl, pH 7.4; 10 mM NaCl; 2.5 mM $MgCl_2$; 1% Igepal CA-630; 0.3 g ml^{-1} sucrose) supplemented with 1 × RNasin Plus RNase Inhibitor, and centrifuged at 450 g for 5 min at 4 °C. The supernatant from this step was designated as the cytoplasmic fraction and the precipitation was designated as the nuclear fraction. The relative mRNA expression levels in the nucleus and cytoplasm were normalized with 45S pre-rRNA and RPS14 respectively and analyzed by RT-qPCR. The values representing the nucleus to cytoplasm ratio were calculated using the $2^{-\Delta Ct}$ method.

RT-qPCR. Total RNA was isolated from PBS-washed cells using the TRIzol reagent. Isolated RNAs were used as templates for reverse transcription based on the manual for HiScript II Q RT SuperMix for qPCR (+gDNA wiper) (Vazyme). Synthesized cDNAs were mixed with ChamQ Universal SYBR qPCR Master Mix (Vazyme) for qPCR in 384-well plates. The reactions were performed in a LightCycler 480 Real-Time PCR System (Roche Applied Science). The house-keeping gene *GAPDH* was used as an internal control. Primers are listed as followed (F, Forward; R, Reverse; sequences are from 5' to 3'):

SOX2-F: CACTGCCCCTCTCACACATG;
SOX2-R: TCCCATTTCCCTCGTTTTTCT;
SOX17-F: GTGGACCGCACGGAATTTG;
SOX17-R: GGAGATTCACACCGGAGTCA;
FOXA2-F: GGAGCAGCTACTATGCAGAGC;
FOXA2-R: CGTGTTCATGCCGTTCATCC;
CXCR4-F: ACTACACCGAGGAAATGGGCT;
CXCR4-R: CCCACAATGCCAGTTAAGAAGA;
GATA4-F: CGACACCCCAATCTCGATATG;
GATA4-R: GTTGCACAGATAGTGACCCGT;
GATA6-F: CTCAGTTCCTACGCTTCGCAT;
GATA6-R: GTCGAGGTCAGTGAACAGCA;
HNF1B-F: ACCAAGCCGGTCTTCCATACT;
HNF1B-R: GGTGTGTCATAGTCGTCGCC;
T-F: CAGTGGCAGTCTCAGGTTAAGAAGGA;
T-R: CGCTACTGCAGGTGTGAGCAA;
MESP1-F: AGCCCAAGTGACAAGGGACAACT;
MESP1-R: AAGGAACCACTTCGAAGGTGCTGA;
TBX6-F: AGCCTGTGTCTTTCCATCGT;
TBX6-R: GCTGCCCGAACTAGGTGTAT;
PDGFRA-F: TGGCAGTACCCCATGTCTGAA;
PDGFRA-R: CCAAGACCGTCACAAAAAGGC;
MIXL1-F: GGCGTCAGAGTGGGAAATCC;
MIXL1-R: GGCAGGCAGTTCACATCTACC;
KDR-F: GGCCCAATAATCAGAGTGGCA;
KDR-R: CCAGTGTCATTTCCGATCACTTT;
PAX6-F: TCGAAGGGCCAAATGGAGAAGAGAAG;

PAX6-R: GGTGGGTTGTGGAATTGGTTGGTAGA;

SOX1-F: GGCGCTGACACCAGACTTGG;

SOX1-R: TCCGCTTCCTCCGTAGGTGA;

TUBB3-F: GGCCAAGGGTCACTACACG;

TUBB3-R: GCAGTCGCAGTTTTCACACTC;

OTX2-F: CATGCAGAGGTCCTATCCCAT;

OTX2-R: AAGCTGGGGACTGATTGAGAT;

DACH1-F: CCCTCTACAATGACTGCACCA;

DACH1-R: GCGGCATGATGTGAGAGTTCT;

JDP2-F: CCCAGCCCGTGAAAAGTGA;

JDP2-R: CGGTGTCGGTTCAGCATCA;

DUSP5-F: TCCTGAGTGTTGCGTGGATG;

DUSP5-R: CTGGTCATAAGCTGGCCTGT;

METTL14-F: AGTGCCGACAGCATTGGTG;

METTL14-R: GGAGCAGAGGTATCATAGGAAGC;

YTHDF2-F: AGCCCCACTTCCTACCAGATG;

YTHDF2-R: TGAGAACTGTTATTTCCCCATGC;

GAPDH-F: AAGGTGAAGGTCGGAGTCAAC;

GAPDH-R: GGGGTCATTGATGGCAACAATA;

qPCR-F for SELECT: ATGCAGCGACTCAGCCTCTG;

qPCR-R for SELECT: TAGCCAGTACCGTAGTGCGTG;

non-m^{6}A-IP-*Spikein*-F: GCTTCAACATCACCGTCATTG;

non-m^{6}A-IP-*Spikein*-R: CACAGAGGCCAGAGATCATTC;

m^{6}A-IP-*Spikein*-F: CGACATTCCTGAGATTCCTGG;

m^{6}A-IP-*Spikein*-R: TTGAGCAGGTCAGAACACTG;

non-m^{6}A-IP-*SOX2*-F: GCCGAGTGGAAACTTTTGTCG;

non-m^{6}A-IP-*SOX2*-R: GGCAGCGTGTACTTATCCTTCT;

m^{6}A-IP-*SOX2*-F: GGCACACTGCCCCTCTCACAC;

m^{6}A-IP-*SOX2*-R: ACCCCTCCCATTTCCCTCGTT;

45S pre-rRNA-F: CCGCGCTCTACCTTACCTAC;

45S pre-rRNA-R: GAGCGACCAAAGGAACCATA;

RPS14-F: GGCAGACCGAGATGAATCCTC;

RPS14-R: CAGGTCCAGGGGTCTTGGTCC.

mRNA stability assay. TRME hESCs were treated with transcription inhibitor actinomycin D (0.01 μg ml^{-1}, MCE) and collected at different times (0, 2 and 4 hours). Total RNA was then isolated by TRIzol reagent. After reverse transcription, the mRNA levels of target transcripts were analyzed by RT-qPCR.

m^6A dot blot. Equal amounts of RNA were dropped on a nylon N^+ membrane (GE Healthcare) at a 70 °C hot stage followed by crosslinking twice under conditions of ultraviolet light at 2,000 × 100 μJ cm^{-2}. The membrane was blocked in PBST buffer containing 5% skimmed milk at room temperature for 1 hour and subsequently incubated with anti-m^6A antibody (E1610S, NEB; 1: 1,000) overnight at 4 °C. After washing three times in PBST buffer, the membrane was incubated with secondary antibody anti-rabbit IgG (7074, CST; 1: 4,000) at room temperature for 1 hour. The membrane was washed three times in PBST buffer. Then the mixture of HRP substrates A and B (Thermo Fisher Scientific) were dropped onto the membrane followed by visualizing in a gel imager. The exposed membrane was placed in a 1% methylene blue solution at room temperature for 10 min, and washed three times in distilled water. Finally, the membrane was photographed.

Statistical analysis. Data were presented as means ± S.E.M quantified from at least three biological repeats unless otherwise stated. The statistical significance of differences was estimated by unpaired, two-tail *t*-test for two-group comparisons using GraphPad Prism 8. For comparisons of multiple groups, one-way ANOVA with a post-hoc Tukey test was used. Samples that were more than two standard deviations from the mean were excluded from the analyses. Differences were considered significant when p-value < 0.05; $*p < 0.05$, $**p < 0.01$; NS, no significant.

References

[1] K. Watanabe, M. Ueno, D. Kamiya, A. Nishiyama, M. Matsumura, T. Wataya, J.B. Takahashi, S. Nishikawa, S. Nishikawa, K. Muguruma, Y. Sasai, *Nat. Biotechnol.* **2007**, *25*, 681.

[2] Y. Xiao, Y. Wang, Q. Tang, L. Wei, X. Zhang, G. Jia, *Angew. Chem. Int. Ed. Engl.* **2018**, *57*, 15995.

[3] D. Kim, J.M. Paggi, C. Park, C. Bennett, S.L. Salzberg, *Nat. Biotechnol.* **2019**, *37*, 907.

[4] Y. Zhang, T. Liu, C.A. Meyer, J. Eeckhoute, D.S. Johnson, B.E. Bernstein, C. Nusbaum, R.M. Myers, M. Brown, W. Li, X.S. Liu, *Genome Biol.* **2008**, *9*, R137.

[5] S. Bae, J. Park, J.S. Kim, *Bioinformatics* **2014**, *30*, 1473.

[6] T. Nojima, T. Gomes, M. Carmo-Fonseca, N.J. Proudfoot, *Nat. Protoc.* **2016**, *11*, 413.

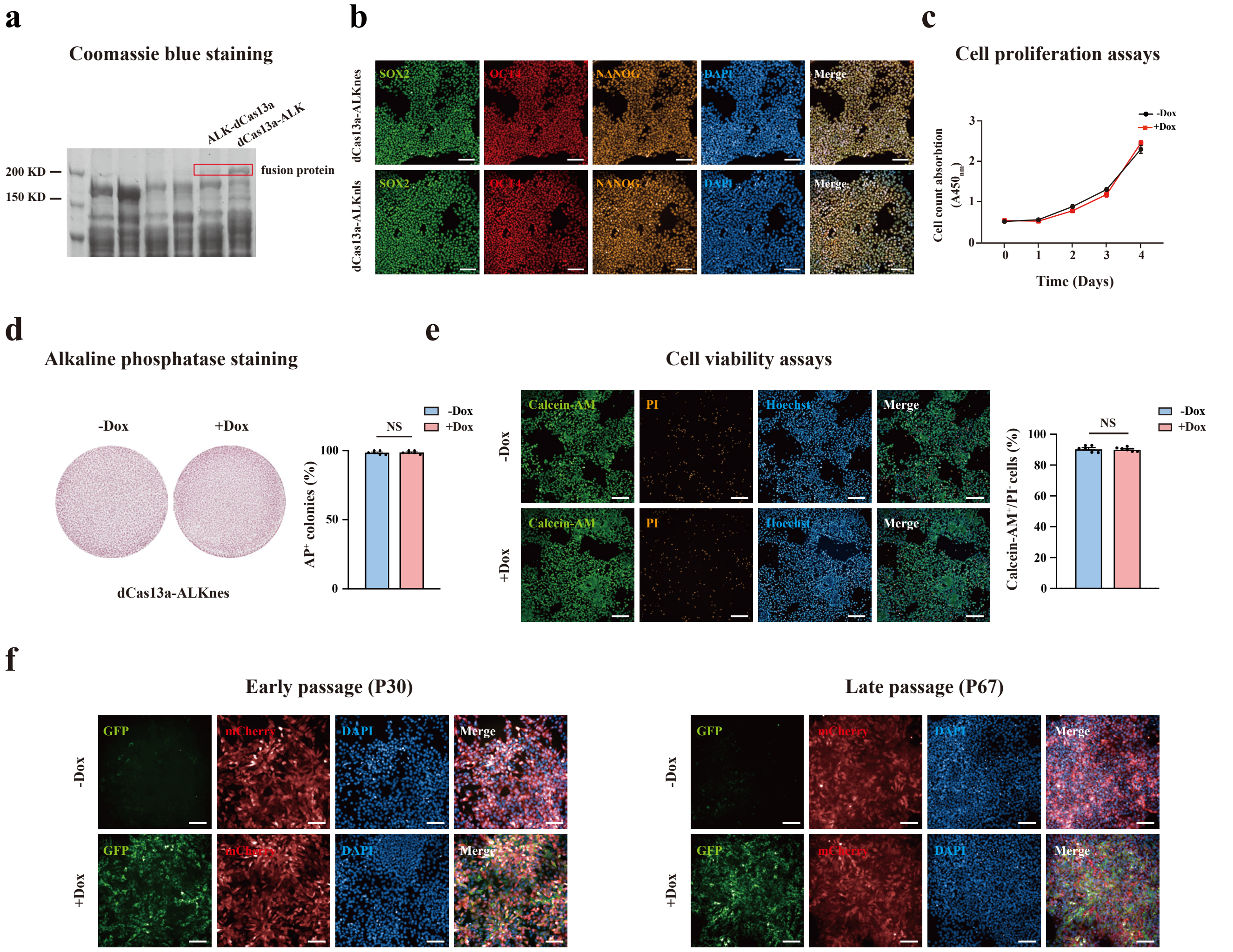

Figure S1. Characterization of the inducible TRME hESCs. a) Coomassie blue staining showing the expression of two fusion proteins in *E. coli*, with ALK tethered to either the C-terminal (dCas13a-ALK) or N-terminal (ALK-dCas13a) of dCas13a. b) Immunostaining analyses of key pluripotency markers, including SOX2, OCT4, and NANOG, in dCas13a-ALKnes (up) and dCas13a-ALKnls (bottom) hESCs. Scale bars, 100 μm. c) Effects of doxycycline on viability and proliferation of dCas13a-ALKnes hESCs detected by the CCK8 assay. $n = 6$ for each group. d) Representative (left) and quantitative (right) results of alkaline phosphatase staining in dCas13a-ALKnes hESCs with or without doxycycline treatment. $n = 6$ for each group. e) Representative (left) and quantitative (right) results of Calcein-AM/PI staining in dCas13a-ALKnes hESCs with or without doxycycline treatment. Scale bars, 200 μm. $n = 6$ for each group. f) Immunostaining analyses of GFP (indicates the expression of TRME editor) and mCherry (indicates the expression of crRNA) expression in dCas13a-ALKnes/S2 hESCs at early (P30) and late (P67) passages with or without doxycycline treatment. Scale bars, 100 μm. Data are shown as the mean ± S.E.M. NS, not significant ($p > 0.05$) using unpaired Student's *t*-test.

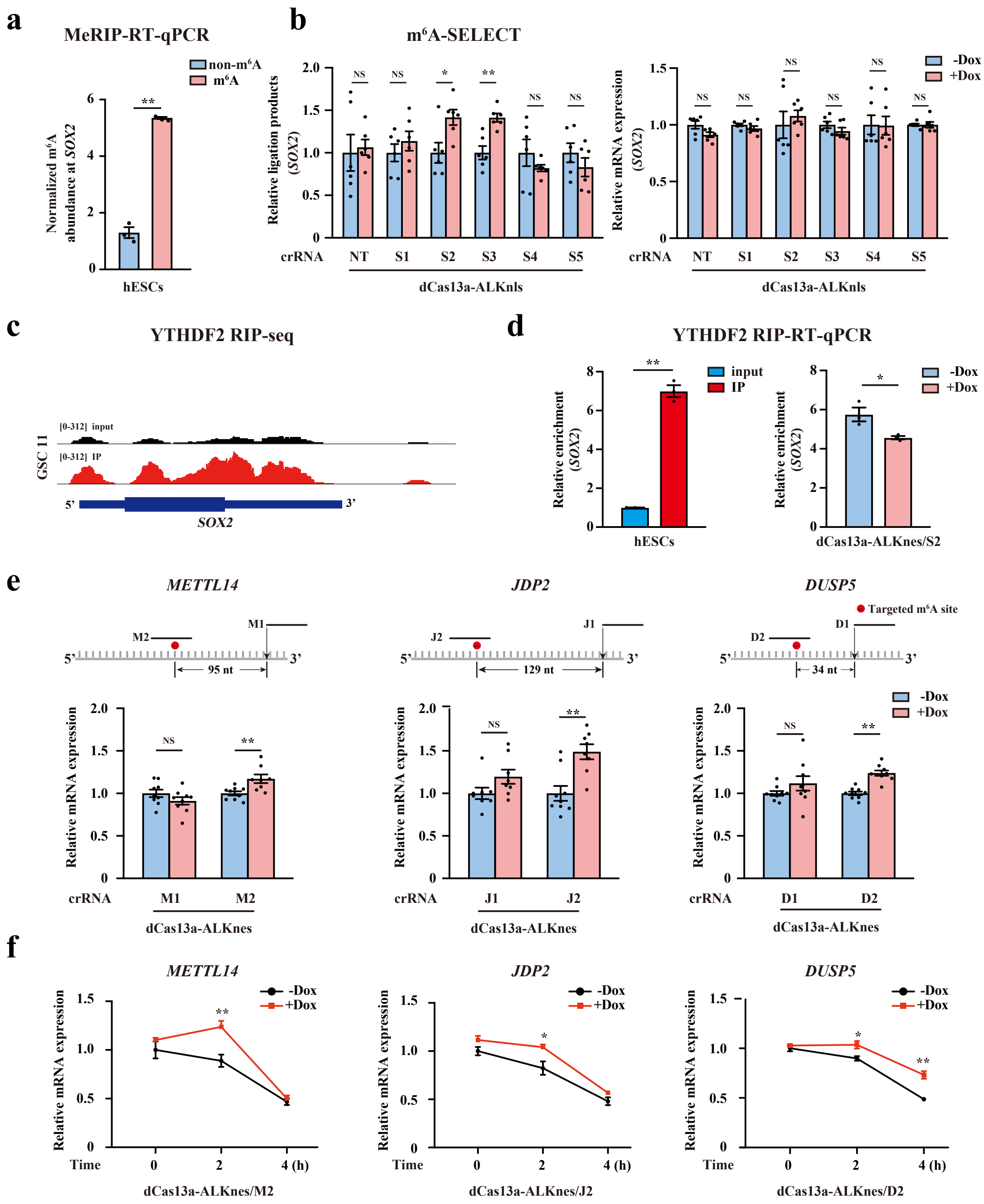

Figure S2. Precise m⁶A demethylation of endogenous transcripts by the cytoplasm-localized TRME editor in hESCs. a) MeRIP-RT-qPCR analysis of m⁶A enrichment on *SOX2* 3'-UTR in hESCs. Primers were designed to span A1398 of *SOX2*. $n = 3$ for each group. b) SELECT (left) and RT-qPCR (right) analyses of m⁶A levels at A1398 and *SOX2* mRNA levels in dCas13a-ALKnls hESCs containing either non-targeting or each *SOX2*-targeting crRNA with or without doxycycline treatment. $n = 6$ for each group. c) YTHDF2 RIP-seq analysis of the binding between YTHDF2 and the 3'-UTR of *SOX2* mRNA in the glioblastoma stem cell line GSC11 (GSE142827). d) YTHDF2 RIP-RT-qPCR analysis of the binding between YTHDF2 and the 3'-UTR of *SOX2* mRNA in wildtype hESCs (left), as well as their binding in dCas13a-ALKnes/S2 hESCs with or without doxycycline treatment (right). $n = 3$ for each group. e) RT-qPCR analysis of the relative mRNA expression of *METTL14*, *JDP2* and *DUSP5* in dCas13a-ALKnes hESCs harboring a crRNA that overlapped or non-overlapped with the targeted m⁶A site within each gene, respectively, in the presence or absence of doxycycline. Targeted m⁶A site within each gene was marked in red. M1 or M2 indicates *METTL14* crRNA1 or 2. J1 or J2 indicates *JDP2* crRNA1 or 2. D1 or D2 indicates *DUSP5* crRNA1 or 2. $n = 8$ for groups M2 (+Dox), J1 (+Dox), J1 (-Dox), and J2 (+Dox). $n = 9$ for other groups. d) Measurement of *METTL14*, *JDP2* and *DUSP5* mRNA decay in dCas13a-ALKnes hESCs containing each m⁶A-targeting crRNA with or without doxycycline treatment. $n = 6$ for each group. Data are shown as the mean ± S.E.M. NS, not significant ($p > 0.05$), $*p < 0.05$, $**p < 0.01$ using unpaired Student's *t*-test.

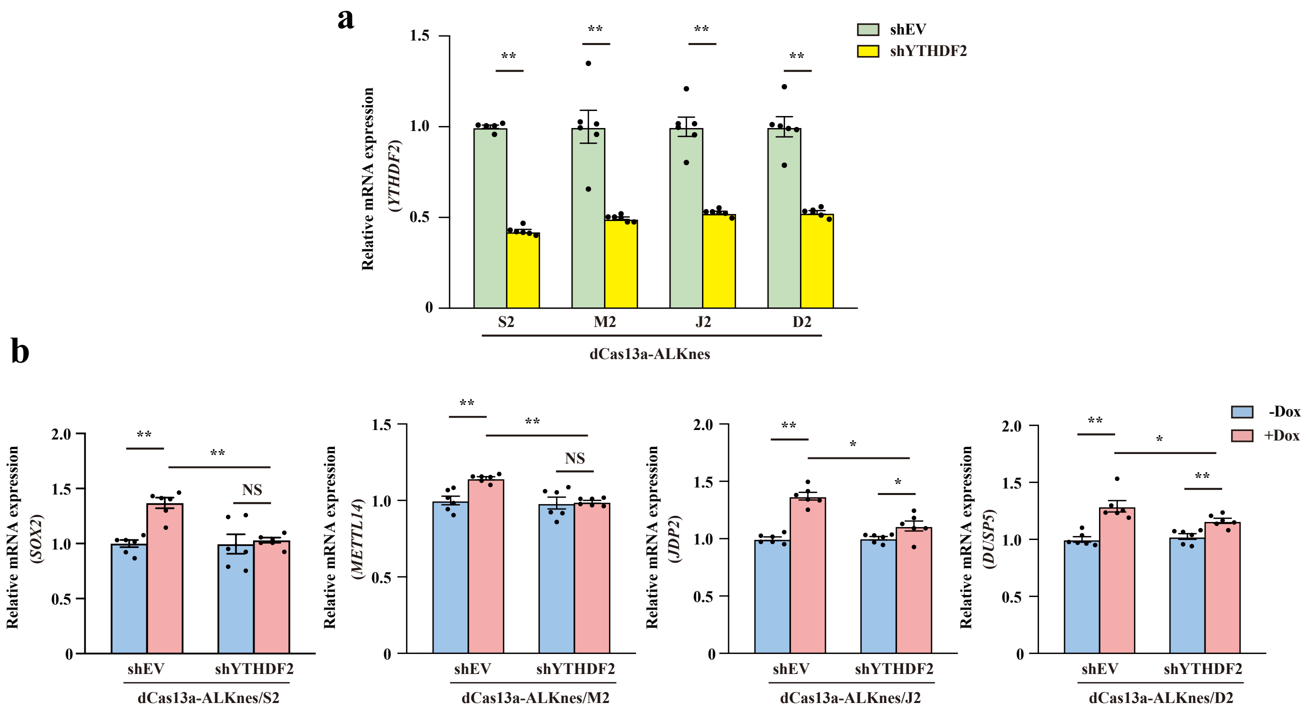

Figure S3. YTHDF2 regulates cytoplasm-localized TRME editor-increased amount and stability of mRNA. a) RT-qPCR analysis showing the knockdown efficiency of YTHDF2 in dCas13a-ALKnes hESCs harboring crRNA that targeting either *SOX2* (S2), *METTL14* (M2), *JDP2* (J2), or *DUSP5* (D2). $n = 6$ for each group. Data are shown as the mean ± S.E.M. $^{**}p < 0.01$ using unpaired Student's t-test. b) RT-qPCR analysis of the corresponding mRNA expression in dCas13a-ALKnes hESCs harboring each gene-targeting crRNA with or without YTHDF2 knockdown, in the presence or absence of doxycycline. $n = 6$ for each group. Data are shown as the mean ± S.E.M. NS, not significant ($p > 0.05$), $^{*}p < 0.05$, $^{**}p < 0.01$ using one-way ANOVA with a post-hoc Tukey test.

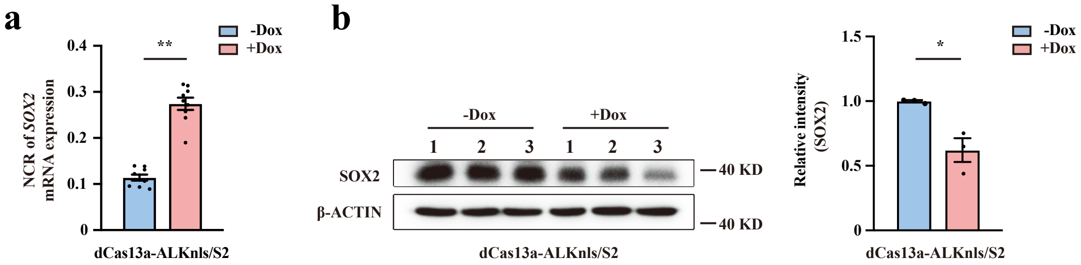

Figure S4. Nucleus-localized TRME editor-induced mRNA demethylation affects nuclei-to-cytoplasm transport of the nascent *SOX2* mRNA. a) Quantification of nucleus to cytoplasm ratio (NCR) of *SOX2* mRNA expression in dCas13a-ALKnls/S2 hESCs with or without doxycycline treatment. $n = 9$ for each group. b) Representative (left) and quantitative (right) results of SOX2 protein expression revealed by western blot analyses in dCas13a-ALKnls/S2 hESCs with or without doxycycline treatment. β-ACTIN was used as a loading control. $n = 3$ for each group. Data are shown as the mean ± S.E.M. NS, not significant ($p > 0.05$), *$p < 0.05$, **$p < 0.01$ using unpaired Student's *t*-test.

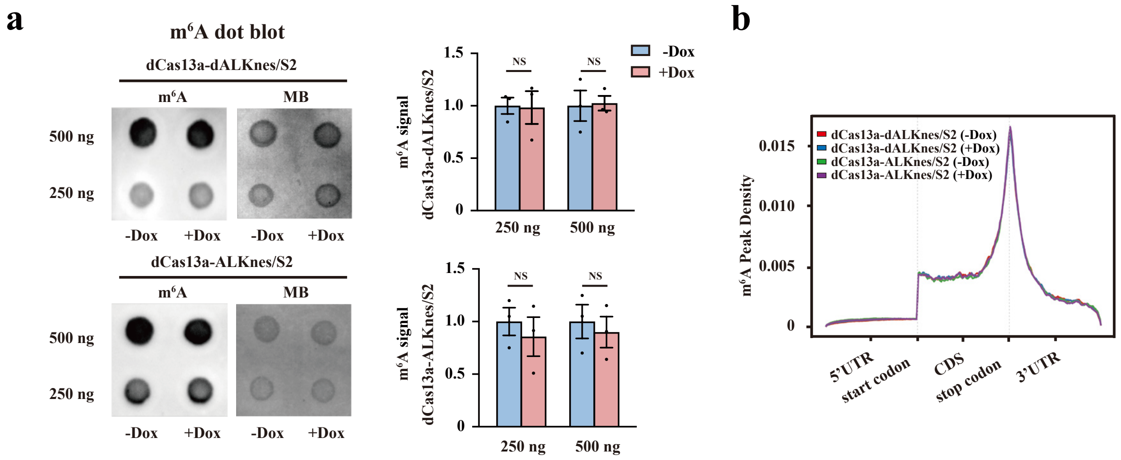

Figure S5. The off-target activity of cytoplasm-localized TRME editors is beyond the detection threshold. a) Representative (left) and quantitative (right) results of global m^{6}A abundance revealed by m^{6}A dot blot in dCas13a-dALKnes/S2 and dCas13a-ALKnes/S2 hESCs with or without doxycycline treatment. Methylene blue (MB) was used as a loading control. n = 3 for each group. b) Metagene profiles of m^{6}A distribution across the transcripts in dCas13a-dALKnes/S2 or dCas13a-ALKnes/S2 hESCs with or without doxycycline treatment. Data are shown as the mean ± S.E.M. NS, not significant ($p > 0.05$) using unpaired Student's t-test.

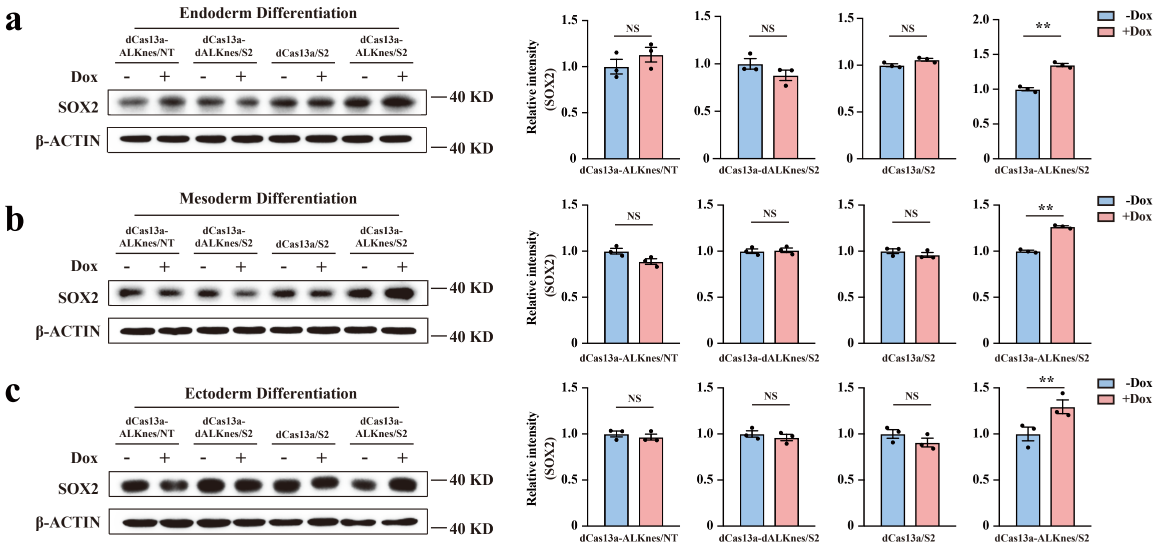

Figure S6. Site-specific demethylation of *SOX2* in the cytoplasm affects the protein level of SOX2 during germ-layer differentiation of hESCs. a,b,c) Representative (left) and quantitative (right) results of SOX2 protein expression revealed by western blot analyses in dCas13a-ALKnes/S2 and other negative control hESCs that underwent either a) endoderm-, b) mesoderm-, or c) ectoderm- differentiation, respectively, in the presence or absence of doxycycline. β-ACTIN was used as a loading control. $n = 3$ for each group. Data are shown as the mean ± S.E.M. NS, not significant ($p > 0.05$), $^{**}p < 0.01$ using unpaired Student's t-test.

Table S1. Off-target prediction of differential m^{6}A loci in dCas13a-ALKnes/S2 with or without Dox.

chrom	start	end	peakname	genename	strand	+Dox-FPKM (A)	-Dox-FPKM (B)	Ratio (A/B)	crRNA pairing
chr2	77320783	77321293	ac_alkbh5_dox_plus_peak_8428	LRRTM4	-	97.78230629	152.6015719	0.64076867	NO
chr19	58083654	58083877	ac_alkbh5_dox_plus_peak_8002	ZNF416	-	67.19976489	98.13537661	0.684765955	NO
chr14	91691238	91691555	ac_alkbh5_dox_plus_peak_4487	C14orf159	+	58.51216426	85.13511917	0.687285868	NO
chr7	73183422	73183842	ac_alkbh5_dox_plus_peak_13231	CLDN3	-	111.7335758	161.5902828	0.691462221	NO
chr7	73245344	73247000	ac_alkbh5_dox_plus_peak_13232	CLDN4	+	258.6512777	366.150967	0.706406103	NO
chr1	2324193	2324479	ac_alkbh5_dox_plus_peak_55	RER1	+	64.10384076	90.29966361	0.709901213	NO
chr6	10626650	10627068	ac_alkbh5_dox_plus_peak_12295	GCNT2	+	66.86118215	93.88255599	0.712178971	NO
chr1	90058360	90058677	ac_alkbh5_dox_plus_peak_668	LRRC8B	+	69.03120502	96.04987804	0.718701642	NO
chr6	26392661	26393970	ac_alkbh5_dox_plus_peak_12366	BTN2A2	+	45.48381033	63.05897788	0.721290003	YES
chr7	1.02E+08	1.02E+08	ac_alkbh5_dox_plus_peak_13418	LOC100630923	+	82.38285923	113.5459525	0.725546419	NO
chr13	52602455	52602729	ac_alkbh5_dox_plus_peak_3915	ALG11	+	53.21678351	73.20429548	0.72696258	NO
chr20	2816061	2816355	ac_alkbh5_dox_plus_peak_9017	FAM113A	-	80.79158174	110.5979566	0.73049796	NO
chr1	2.13E+08	2.13E+08	ac_alkbh5_dox_plus_peak_1312	FLVCR1-AS1	-	70.19000223	95.99752907	0.731164676	NO
chrX	1.14E+08	1.14E+08	ac_alkbh5_dox_plus_peak_15350	HTR2C	+	74.66640543	101.9230578	0.732576191	NO

chr4	559454 50	559463 30	ac_alkbh5_dox_plus_ peak_11059	KDR	-	119.364 5464	160.21528	0.7450 25983	NO
chr5	522320 5	522374 3	ac_alkbh5_dox_plus_ peak_11516	ADAMTS1 6	+	128.775 307	172.57874 02	0.7461 82913	NO
chr8	705707 40	705714 45	ac_alkbh5_dox_plus_ peak_14055	SULF1	+	79.6582 671	106.51938 9	0.7478 28803	NO
chr11	1.3 E+ 08	1.3E+0 8	ac_alkbh5_dox_plus_ peak_2926	ST14	+	74.6664 0543	99.829503 11	0.7479 39268	NO
chr19	532688 26	532692 94	ac_alkbh5_dox_plus_ peak_7881	ZNF600	-	69.9858 2479	93.247880 24	0.7505 3529	NO
chr9	274551 75	274556 44	ac_alkbh5_dox_plus_ peak_14370	MOB3B	-	71.1713 822	94.526456 37	0.7529 25529	NO
chr7	1.5 E+ 08	1.5E+0 8	ac_alkbh5_dox_plus_ peak_13647	LRRC61	+	67.3716 313	89.361447 8	0.7539 22782	NO
chr10	537841 59	537853 21	ac_alkbh5_dox_plus_ peak_1722	PRKG1	+	79.9951 3428	106.04678 36	0.7543 38148	NO
chr19	334608 71	334611 75	ac_alkbh5_dox_plus_ peak_7506	CEP89	-	56.2078 9078	74.349190 24	0.7559 98695	NO
chr11	685042 51	685053 53	ac_alkbh5_dox_plus_ peak_2622	MTL5	-	92.4969 8675	122.30467 24	0.7562 83345	NO
chr20	374003 27	374007 71	ac_alkbh5_dox_plus_ peak_9221	ACTR5	+	74.2301 5228	97.756995 38	0.7593 33406	NO
chr12	1.3 3E +0 8	1.33E+ 08	ac_alkbh5_dox_plus_ peak_3737	ZNF605	-	52.6425 0167	69.084926 99	0.7619 96921	NO
chr1	1.5 6E +0 8	1.56E+ 08	ac_alkbh5_dox_plus_ peak_976	MSTO1	+	88.2296 6073	115.69644 4	0.7625 96132	NO
chr3	727995 57	727999 70	ac_alkbh5_dox_plus_ peak_10350	SHQ1	-	77.2634 978	101.16452 35	0.7637 41034	NO
chr16	252582 58	252586 72	ac_alkbh5_dox_plus_ peak_5466	ZKSCAN2	-	64.9867 6058	84.193556 84	0.7718 73324	NO
chr1	1.9 3E +0 8	1.93E+ 08	ac_alkbh5_dox_plus_ peak_1173	RGS2	+	83.6263 7409	108.33394 3	0.7719 31416	NO

chr17	17696610	17696860	ac_alkbh5_dox_plus_peak_6073	RAI1	+	69.13335706	89.42274955	0.773107038	NO
chrX	1.08E+08	1.08E+08	ac_alkbh5_dox_plus_peak_15337	IRS4	-	80.0817711	103.5274303	0.773531912	NO
chr7	1.52E+08	1.52E+08	ac_alkbh5_dox_plus_peak_13686	MLL3	-	50.79607122	65.09774343	0.780304639	NO
chr18	21735699	21735963	ac_alkbh5_dox_plus_peak_6822	CABYR	+	60.74746042	77.71308314	0.781688977	NO
chr7	51096605	51096918	ac_alkbh5_dox_plus_peak_13169	COBL	-	109.1936732	139.2778848	0.783998646	NO
chr5	1.47E+08	1.47E+08	ac_alkbh5_dox_plus_peak_12041	LOC153469	+	88.99995636	113.3732222	0.785017437	NO
chr8	1719098	1719709	ac_alkbh5_dox_plus_peak_13752	CLN8	+	79.59536422	101.3289117	0.785514843	NO
chr10	53782781	53783847	ac_alkbh5_dox_plus_peak_1721	PRKG1	+	68.1864531	86.74522512	0.786054253	NO
chr4	1.64E+08	1.64E+08	ac_alkbh5_dox_plus_peak_11400	NPY1R	-	121.1753268	153.9123232	0.787301005	NO
chr16	3349090	3349450	ac_alkbh5_dox_plus_peak_5281	TIGD7	-	67.17908168	85.25001137	0.788024313	NO
chr7	1.31E+08	1.31E+08	ac_alkbh5_dox_plus_peak_13525	PODXL	-	185.2415201	234.6519428	0.78943101	NO
chr7	12727791	12728712	ac_alkbh5_dox_plus_peak_13029	ARL4A	+	179.1345866	226.8754563	0.789572347	NO
chr6	42196000	42196389	ac_alkbh5_dox_plus_peak_12477	TRERF1	-	74.51324358	94.33710049	0.789861499	NO
chr12	7842483	7843300	ac_alkbh5_dox_plus_peak_3045	GDF3	-	113.7339134	143.9840831	0.789906155	NO
chr4	1244166	1244850	ac_alkbh5_dox_plus_peak_10851	C4orf42	+	70.19732131	88.841357	0.790142381	NO

chr20	3146143	3147006	ac_alkbh5_dox_plus_peak_9028	ProSAPiP1	-	165.510532	209.4319889	0.79028296	NO
chr1	2.39E+08	2.39E+08	ac_alkbh5_dox_plus_peak_1491	LOC339535	-	80.47548219	101.79402	0.790571806	NO
chr1	2.26E+08	2.26E+08	ac_alkbh5_dox_plus_peak_1388	LEFTY1	-	514.9533896	651.1876356	0.790791104	NO
chr10	1.03E+08	1.03E+08	ac_alkbh5_dox_plus_peak_1983	C10orf2	+	197.7109164	249.1354737	0.793587976	NO
chr3	1.81E+08	1.81E+08	ac_alkbh5_dox_plus_peak_10714	**SOX2**	+	246.7550278	309.4702428	0.797346541	**target**
